# How Binding Site Flexibility Promotes RNA Scanning by TbRGG2 RRM: A Molecular Dynamics Simulation Study

**DOI:** 10.1021/acs.jcim.4c01954

**Published:** 2025-01-13

**Authors:** Toon Lemmens, Jiří Šponer, Miroslav Krepl

**Affiliations:** † Institute of Biophysics of the Czech Academy of Sciences, Kralovopolska 135, 612 00 Brno, Czech Republic; ‡ National Centre for Biomolecular Research, Faculty of Science, Masaryk University, Kamenice 5, 625 00 Brno, Czech Republic

## Abstract

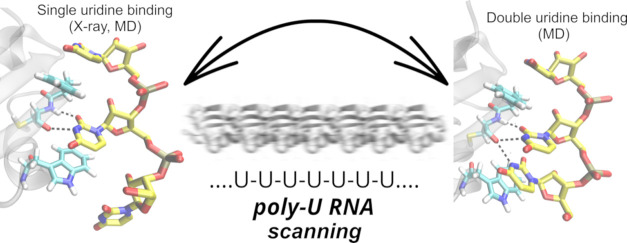

RNA recognition motifs (RRMs) are a key class of proteins that primarily bind single-stranded RNAs. In this study, we applied standard atomistic molecular dynamics simulations to obtain insights into the intricate binding dynamics between uridine-rich RNAs and TbRGG2 RRM using the recently developed OL3-Stafix AMBER force field, which improves the description of single-stranded RNA molecules. Complementing structural experiments that unveil a primary binding mode with a single uridine bound, our simulations uncover two supplementary binding modes in which adjacent nucleotides encroach upon the binding pocket. This leads to a unique molecular mechanism through which the TbRGG2 RRM is capable of rapidly transitioning the U-rich sequence. In contrast, the presence of non-native cytidines induces stalling and destabilization of the complex. By leveraging extensive equilibrium dynamics and a large variety of binding states, TbRGG2 RRM effectively expedites diffusion along the RNA substrate while ensuring robust selectivity for U-rich sequences despite featuring a solitary binding pocket. We further substantiate our description of the complex dynamics by simulating the fully spontaneous association process of U-rich sequences to the TbRGG2 RRM. Our study highlights the critical role of dynamics and auxiliary binding states in interface dynamics employed by RNA-binding proteins, which is not readily apparent in traditional structural studies but could represent a general type of binding strategy employed by many RNA-binding proteins.

## Introduction

**Figure 1:**
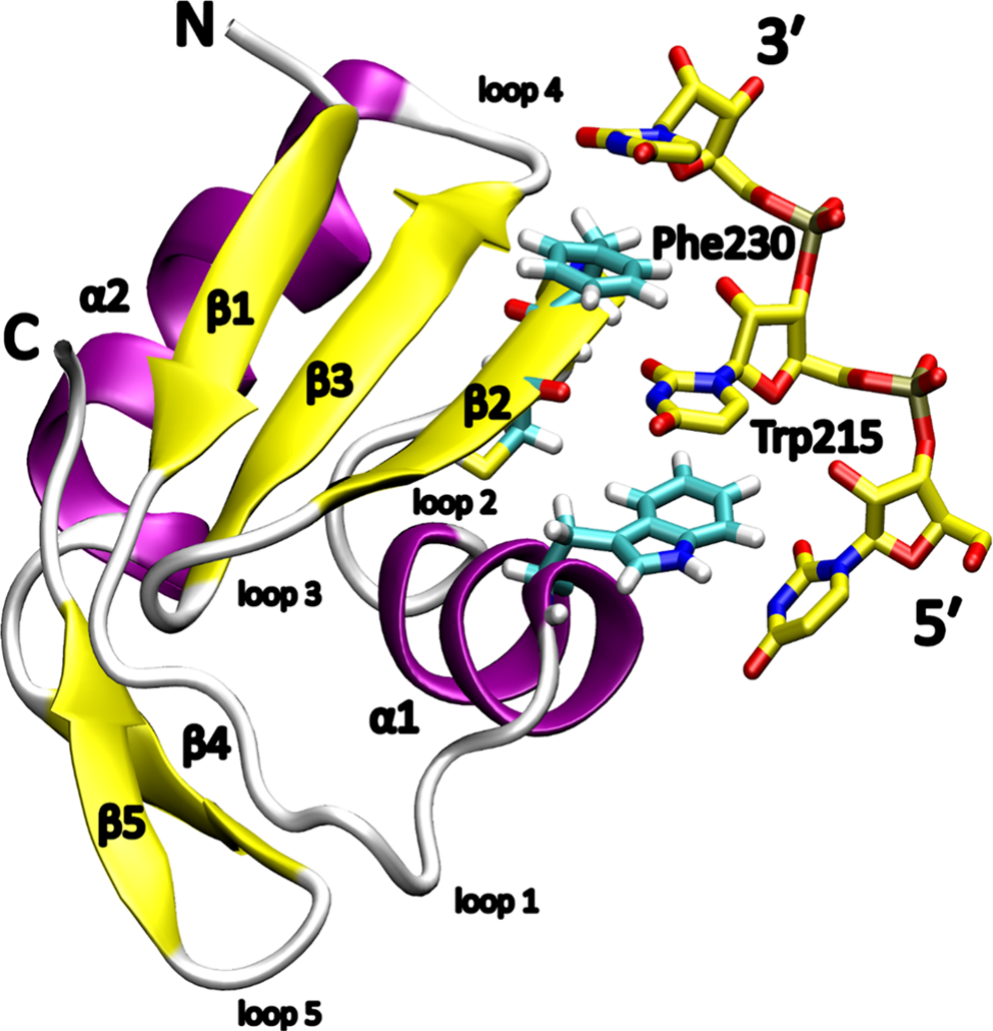
Structure of TbRGG2 RRM complexed with poly(U) single-stranded RNA (ssRNA). The chain termini and protein secondary structure are annotated, and the α-helices, β-sheets, and loops are shown in purple, yellow, and white, respectively. The RNA and binding pocket amino acids are displayed as sticks, and their carbon atoms are colored yellow and cyan, respectively.

The RNA recognition motif (RRM) is a common RNA-binding protein domain in eukaryotes, capable of recognizing various RNA sequences with high fidelity.^1^ This makes the RRM-containing proteins a ubiquitous feature of RNA metabolism, including expression, splicing, editing, and stabilization.^2^ Up to 1% of human genes include RRMs,^3^ and their widespread utilization in eukaryotic evolution stands in stark contrast to their relatively simple and conserved topology. Namely, a single RRM domain is typically composed of ∼90 amino acids with a consensual secondary structure of β_1_α_1_β_2_β_3_α_2_β_4_,^4^ where two α-helices fold against one side of the antiparallel β-sheet surface (Figure 1). While its structure shows limited variations among the different proteins, the RRM’s specificity for different RNA sequences can be astoundingly tuned via mutations of its surface-exposed amino acids.^4−11^ This ability is further enhanced by the diverse modes of protein–RNA recognition that the RRMs can facilitate. Namely, the exposed β-sheet surface of RRM is the most common binding site for RNAs,^12^ but in principle, any part of the domain can be tuned for RNA recognition, including the α-helices, the loops, and the terminal chains.^9,10,13−18^ Usually, two to five nucleotides are directly recognized by a single RRM.^3^ The RNA-binding proteins can contain multiple RRM domains or act on a single substrate as multimers, further increasing the specificity of RRM-RNA recognition. The widespread role of RRM domains in RNA metabolism made them a staple of protein–RNA structural biology, and numerous structures of RRM protein–RNA complexes were determined by X-ray crystallography and NMR spectroscopy.^2^ A limitation of these studies is that they basically provide a static ensemble-averaged picture of the bound protein–RNA complex in equilibrium. In reality, the RRM-RNA interaction, even at equilibrium, would be more accurately described as a dynamic ensemble of conformations sampled with different lifetimes and populations.^11,19−22^ Likewise, the structural details of the binding process (binding pathways) cannot be inferred from structural studies of fully bound complexes.^23^

Molecular dynamics (MD) simulations are a computational method for studying the movements of biomolecules using a carefully calibrated set of empirical potentials commonly known as the force fields (ffs). Explicit-solvent standard MD can be used to routinely investigate thermal fluctuations of the entire protein–RNA complex on a multiple-microsecond scale with effectively infinite spatial and temporal resolution.^24−26^ This level of detail is currently inaccessible for experimental methods, and the simulations thus fill an important gap in our knowledge of biomolecular systems. However, the MD is limited by the affordable length of the calculation and adequacy of the utilized ffs.^24^ The latter can be particularly challenging for the description of the protein–RNA complexes as the ffs are generally optimized for simulations of isolated proteins and nucleic acids. One such ff issue is spurious overcompaction of single-stranded RNA (ssRNA) molecules with the standard RNA ffs.^27−29^ The overcompaction is primarily caused by the overestimated attraction of van der Waals interactions (not only the base stacking) along the RNA chain.^23^ As a result, the RNA–RNA interactions tend to overwhelm the intermolecular interactions and disrupt protein–ssRNA interfaces.^30,31^ We have recently tackled this problem by developing a ff modification called Stafix, which eliminates the overcompaction and significantly improves the ff performance in protein–ssRNA simulations. Stafix allows simulations of the binding process of protein–ssRNA complexes starting from the fully unbound state.^23^ It even facilitates predictions of protein–RNA interfaces in complexes where experimental structures are unavailable.^32^ Stafix has been implemented through a nonbonded fix (NBfix) modification of the Lennard-Jones (LJ) matrix, targeting specific pairwise interactions among the RNA atoms, i.e., modifying the standard Lorentz–Berthelot combination rules of the ff.^33^ Importantly, it does not alter the LJ interactions with the other components of the system, such as water, ions, or proteins. Furthermore, Stafix has been carefully constructed to preserve intramolecular RNA hydrogen bonds and maintain the syn/anti balance of the *N*-glycosidic angle. Stafix was not created or extensively tested for the simulations of structured RNA motifs. Still, our OL3-Stafix simulations of A-RNA duplexes demonstrated that Stafix introduces only negligible structural dynamics changes compared to simulations with the standard OL3 RNA ff (Figure S12 in ref (23)). No deformations of the backbone or loss of helicity were observed. Therefore, we suggest that Stafix could also be applied in protein–RNA systems containing an RNA stem–loop, another common class of RNAs regularly recognized by RRMs. In summary, Stafix is a goal-specific modification of the standard RNA simulation ff; however, such targeted adjustments are justified, given the major challenges in developing universally applicable RNA ffs.^34,35^ See the Supporting Information for additional comments on the Stafix ff modification.

**Figure 2:**
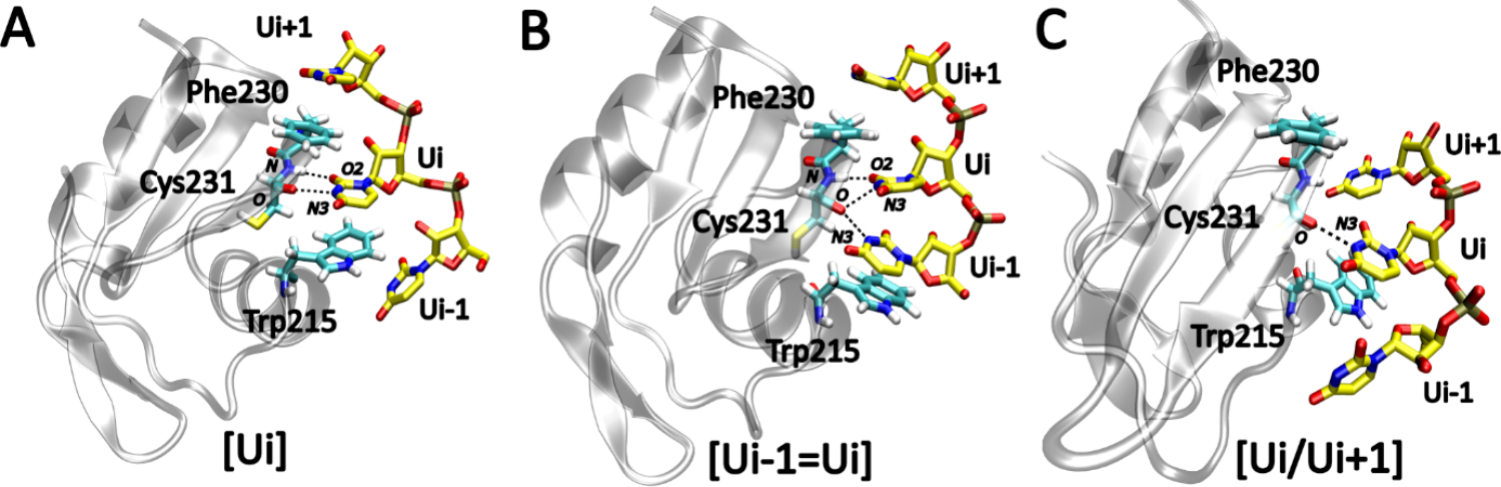
Representative structures of the three dominant states of the TbRGG2 RRM-poly(U) binding interface as observed in MD simulations. (A) Single uridine (observed also in the experiment), (B) the vertical state, and (C) the horizontal state. The H-bonds characterizing each state are indicated with black dashed lines, and the protein is shown as a transparent gray ribbon. To annotate the three states, we use symbolic representations of binding pocket structure, where “[” and “]” refer to the clamp formed by the Trp215 and Phe230 side chains, respectively, while “=” and “/” indicate vertical and horizontal arrangements of the uridines, respectively. The “i–1,” “i+1,” and “i±1” indices indicate upstream, downstream, and either of the nucleotides, respectively. This annotation is utilized throughout the study. Where relevant for the description, “i” is replaced with a specific uridine residue number.

The TbRGG2 is a protein responsible for kinetoplastid RNA (kRNA) editing in the kinetoplastid protists, such as the *Trypanosoma brucei*.^36^ During the kRNA editing, specific uridine insertions or deletions are performed on the mitochondrial mRNAs, the extent of which varies among the transcripts, ranging between a few to hundreds of uridines.^37,38^ The TbRGG2 alters the mRNA structure, a process that was shown as essential for successful kRNA editing.^39^ The TbRGG2 is composed of two domains, the N-terminal glycine-rich region and C-terminal RRM;^40^ the latter is studied in this work. The TbRGG2 RRM (Figure 1) stands out among the other RRMs in several ways. First, it exhibits a rare multimodal binding by specifically recognizing both the U-rich and G-rich RNAs via separate binding sites. Second, the binding site for the U-rich sequences consists of a single binding pocket recognizing one uridine (Figures 1 and 2A). The binding site of the G-rich sequences is currently unknown.^41^ Note that TbRGG2 is also sometimes referred to as RESC13 in the literature.^42,43^

Here, we used MD simulations to study the μs-scale equilibrium dynamics of the complex between TbRGG2 RRM and poly(U) ssRNA. We show that the dynamics of the binding site is characterized by large-scale conformational fluctuations, including fully reversible loss of the specific interactions. The key observation is that the main binding mode (shown by experiments) can be transiently replaced with two alternative binding modes in which two uridines simultaneously occupy the pocket. Strikingly, the newly observed binding modes are highly selective for uridines as well. Such structural dynamics of the interface allows smooth sliding of the RNA along the protein, and we sometimes observed complete spontaneous shift of the poly(U) sequences all the way to the strand termini. An encounter with non-native cytidines tends to cause either a swift transition back to U-rich regions or disengagement of the RNA from the binding pocket, followed by another binding attempt elsewhere on the sequence. We also carried out spontaneous binding simulations starting from the unbound state. We observed the successful formation of the native binding state as indicated by the experiments, followed by the characteristic equilibrium dynamics of the bound state. In other words, these simulations revealed the same behavior as simulations initiated from the bound state, confirming that the observed equilibrium dynamics is quite converged and not biased by the starting structure. We suggest that dynamic binding mechanisms such as the one employed by TbRGG2 RRM could constitute so far underappreciated element of protein–RNA recognition, largely invisible to conventional structural experiments due to its transient nature,^44^ but with potentially significant importance and utility in tuning protein–RNA interactions.

## Methods

### Selection and Preparation of Initial Structures

We have used the X-ray structure of the *T. brucei* TbRGG2 RRM domain complexed with the 5′-UUU-3′ (3U) RNA (chains B/K; PDB: 6E4P) as the starting structure, with the individual uridines numbered from U1 to U3.^41^ An additional RRM domain (chain A) was used in a few simulations (see the Supporting Information). In some simulations, we have extended the RNA chain at both ends using Pymol to obtain complexes with bound 5′-UUUUU-3′ (5U) RNA, adding uridines U0 and U4 at the 5′- and 3′-ends, respectively. As starting structures for some of the 5U complex simulations, we also used simulation snapshots in which two uracils simultaneously occupy the binding pocket. By replacing individual nucleotides with cytidines in the 5U complex, we obtained starting structures with bound RNA sequences of 5′-UCUUU-3,′ 5′-UUUCU-3,′ 5′-UUCUU-3,′ 5′-CCUCC-3,′ or 5′-CCCCC-3.′ In some simulations, the TbRGG2 RRM was modified by replacing the K219 and K232 residues with alanines. Simulations of the free protein (chain B; PDB 6E4P) were performed by removing the RNA from the complex structure.

The initial structures for the spontaneous binding simulations from the unbound state^23^ were prepared by placing the TbRGG2 RRM protein and the 5′-UUUU-3′ RNA ∼20 Å apart. This distance was shown to be sufficient for randomizing the ssRNA’s internal structure and the first spontaneous point of contact between the two biomolecules.^23^ For the spontaneous binding simulations, the initial RNA structure was prepared with Nucleic Acid Builder and corresponded to an A-RNA helix with the complementary strand removed. Poly(U) sequence of four nucleotides was selected to increase sampling efficiency compared to longer sequences and to reduce off-pathway binding.^23^ The uridines in this case were counted from U1 to U4. Lastly, initial coordinates for the simulations of the putative RRM homodimer with and without bound RNA were obtained from chains A/B/K and A/B (or A/D) of the 6E4P structure, respectively (Supporting Information).

### System Building, Force Fields, and Simulation Protocol

We utilized the tLeap program of AMBER 20^45^ to generate the initial simulation files. The RNA was described with the OL3 ff,^46^ and we additionally applied the recently developed Stafix potential,^23^ which eliminates spurious RNA self-interactions via extensive rescaling of the intra-RNA van der Waals interactions (see also Introduction and the Supporting Information). The used ff thus could be abbreviated as OL3-Stafix. Stafix greatly improves the accuracy of the simulations of bound protein–ssRNA complexes and is indispensable for simulations of spontaneous binding of ssRNA to proteins from the unbound state.^23^ The applied Stafix factor for all bound simulations and most of the spontaneous binding simulations was 0.5. A factor of 0.1 was also tested for some of the spontaneous binding simulations. Stafix was implemented by modifying the AMBER topology files using the published scripts (https://zenodo.org/records/7273724). The RRM was described by the ff12SB ff;^47^ note that for RRM protein–RNA complexes, we prefer to use the ff12SB version over the ff14SB for reasons explained elsewhere.^23^ The biomolecules were immersed in an octahedral box of SPC/E water molecules^48^ with a minimal distance of 12 Å between the solute and the edge of the simulation box. Some of the binding simulations were also performed with the OPC water model^49^ or with the application of the gHBfix19 RNA ff modification.^50^ Physiological excess-salt ion concentration of 0.15 M was obtained by adding KCl ions^51^ at random positions around the solute. The equilibration of the systems was performed using the standard protocol^52^ in the pmemd.MPI program of AMBER 20. Afterward, we ran production simulations of varying lengths (2–10 μs) using the GPU-enabled pmemd.cuda program.^53^ In all cases, multiple parallel simulations of each system were carried out. SHAKE^54^ and hydrogen mass repartitioning^55^ were applied in all simulations, allowing a 4 fs integration step. Long-range electrostatics was handled by the particle mesh Ewald^56^ in a periodic boundary conditions setup. The distance cutoff for the calculation of the nonbonded Lennard-Jones (LJ) interactions was 9 Å. Langevin thermostat and Monte Carlo barostat^45^ were used to regulate the production simulations.

### Analyses

We have used cpptraj^57^ and VMD^58^ to analyze and visualize the trajectories, respectively. Graphs were prepared with Gnuplot and Excel, and molecular figures were prepared with Inkscape. To monitor the state of the TbRGG2 RRM binding pocket, we visually inspected every trajectory. The state of the pocket was manually annotated based on the number of occupying nucleotides and their mutual orientation and identity. The resulting data are displayed using horizontal bar graphs, where individual states are distinguished by different colors, and a table lists the relative populations of each state. Apart from visual analysis, we also separately evaluated the state of the pocket using an automated method, where the distinction is made based on the H-bonds formed between the nucleotides and the Cys231(O) and Cys231(N) atoms, with a cutoff distance of 3.5 Å between heavy atom donors and acceptors and an acceptor–hydrogen-donor angle of 135°. A second criterion was the distance between Trp215(CE3) and Phe230(CG) atoms, with distances below and above 9.0 Å corresponding to the single state and the vertical/horizontal states, respectively. The automated method was used to quickly evaluate the approximate state of the pocket in each simulation; however, due to the enormous complexity of the binding pocket dynamics, we ultimately used manual annotation to produce the graphs below.

## Results

Below, we present the results of multiple-microsecond explicit-solvent MD simulations of the TbRGG2–poly(U) complex (Table 1). We also explore complexes involving RNA sequences with varying content of cytidines or mutated protein domains. Finally, we present the results of spontaneous binding simulations, where we have studied the entire association process of the TbRGG2-poly(U) complex, starting from the unbound state. The number of conducted simulations is 60, with a cumulative length of 524 μs. The results below describe the key observations derived from our analyses of simulated complexes. Simulations of isolated RRM and its putative homodimers are described in the Supporting Information.

**Table 1:** List of Simulations

simulation name	RNA sequence	length (μs)^a^
Simulations of Bound Complexes		
MD1	5′-UUU-3′	5/5
MD2	5′-UUUUU-3′	5/5/10/10
MD3^b^	5′-UUUUU-3′	10/10/2/2/2/2
MD4^b^	5′-UUUUU-3′	10/10/2/2/2/2
MD5	5′-UUCUU-3′	10/10/10/10
MD6	5′-UUUCU-3′	10/10/10/10
MD7	5′-UCUUU-3′	10/10/10/10
MD8	5′-CCUCC-3′	10/10/10/10
MD9	5′-CCCCC-3′	10/10/10/10
MD10^c^	5′-UUUUU-3′	5/5/5/5
MD11^d^	5′-UUUUU-3′	5/5/5/5
MD12^c^^,^^d^	5′-UUUUU-3′	5/5/5/5
Spontaneous Binding Simulations		
MD13^e^	5′-UUUU-3′	10/10/10/10
MD14^e^^,^^f^	5′-UUUU-3′	10/10/10/10
MD15^g^	5′-UUUU-3′	10/10/10/10/10/10

a Simulation replicates (henceforth denoted as R*X*, where *X* is the number of the replicate; e.g., MD1|R2 refers to the second simulation replicate of the MD1 system) and their respective lengths.

b Snapshots taken from simulation MD2|R1 at 149 and 3904 ns were used as the starting structures for MD3 and MD4, respectively.

c Lys219 was replaced with alanine.

d Lys232 was replaced with alanine.

e The OPC water model was applied.

f gHBfix19 ff modification^50^ was added to the RNA OL3-Stafix ff.

g Stafix factor 0.1^23^ was applied.

### Temporary Accommodation of Two Uridines at the Binding Site Is the Hallmark of the TbRGG2 RRM Complex Dynamics

The experimental X-ray structure (PDB: 6E4P) shows one uridine stacked between the binding pocket residues Trp215 and Phe230 (Figure 2A), with the base specifically recognized by U2(N3)–Cys231(O) and Cys231(N)–U2(O2) H-bonds.^41^ We call this arrangement the single uridine state. All of the MD simulations initiated from this structure revealed significant (sub) μs-scale dynamics involving conformational transitions and temporary interruptions of the binding. Importantly, we have regularly observed two additional binding states in which two uridines simultaneously occupy the pocket. Based on the mutual orientation of the two bases within the pocket, we named them as the vertical and horizontal states. The vertical state is formed by two uridines sandwiched between Trp215 and Phe230, giving rise to a quadruple stack (Figure 2B). Note that it may involve both upstream and downstream uridine with respect to the originally bound single uridine. In the horizontal state, the first uridine is sandwiched between Trp215 and Phe230, while the second (downstream) uridine interacts with a hydrophobic patch on the RRM’s surface. This places both bases in roughly the same plane (Figures 2C and S1). Instances in which nonconsecutive uridines formed either the horizontal or vertical states also appeared, albeit rarely. The populations of the individual binding pocket states varied significantly, even among individual replicates of the same system (Supporting Information), indicating that quantitatively converged sampling was not achieved. Nevertheless, the same binding states were consistently observed across all trajectories. In conclusion, the hallmark of dynamics of the TbRGG2–poly(U) complex on a microsecond time scale are frequent exchanges between the single uridine state and the vertical and horizontal states. These are sometimes temporarily interrupted by an unbound state where no uridines are present within the binding pocket, even though the RNA remains attached to the protein surface in the proximity of the binding pocket.

### TbRGG2 Specifically Recognizes Uridines in All Three Binding States

The single uridine state involves the Cys231(O)–Ui(N3) and Cys231(N)–Ui(O2) H-bonds. These interactions also align the uridine base for optimal stacking with the Trp215 and Phe230 aromatic side chains (Figure 2A). Such an arrangement is not sterically feasible for larger purine bases, while cytidine lacks compatible H-bond donors and acceptors. Strikingly, the specificity for uridines is maintained for the vertical and horizontal binding states observed in our simulations. Namely, in the vertical state, the second uridine Ui–1 is also engaged in the Cys231(O)–Ui–1(N3) H-bond, resulting in bifurcated H-bonding (Figure 2B). In the horizontal state, the uridine Ui located deeper in the binding pocket is recognized similarly to the single uridine state, while the additional downstream uridine Ui+1 is recognized by Val228(O)–Ui+1(N3) H-bond. There is also a fluctuating H-bond between Lys219(NZ) and Ui+1(O4) (Figure S2). In the vertical state, the distance between the aromatic rings of Trp215 and Phe230 increases by ∼1.0 Å on average compared with the single uridine state, reflecting the accommodation of the additional nucleotide. The distance similarly increases by ∼0.5 Å for the horizontal state. For details of the H-bond populations in all three states, see the Supporting Information.

### Accommodation of Two Uridines by TbRGG2 RRM Facilitates Fast Diffusion of the Bound RNA

**Figure 3:**
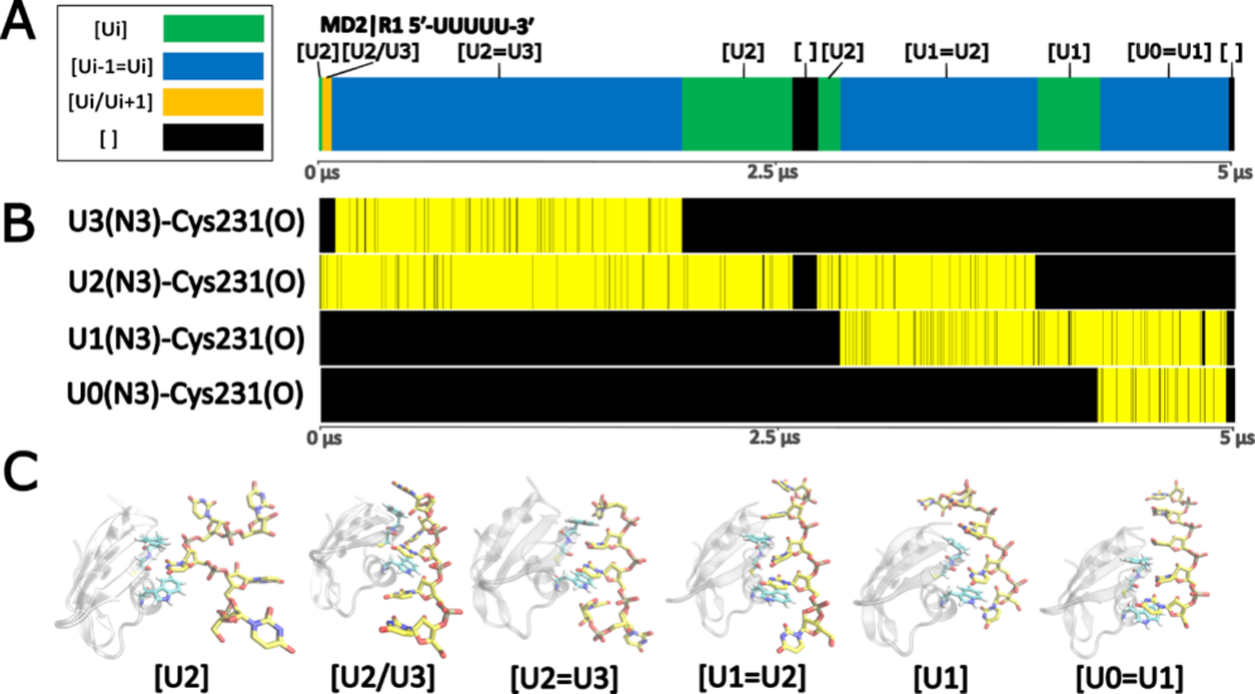
Example of TbRGG2 RRM diffusion along the 5′-UUUUU-3′ RNA. A time-development analysis of the MD2|R1 trajectory (see Figure S3 for the description of all other trajectories). (A) Graph of the binding pocket occupancy. The different binding states are color-coded according to the legend on the left. See also the legend of Figure S3. “[]” indicates no uridine is present in the binding pocket. (B) Time-development of the characteristic H-bond formed between the N3 atoms of the occupying uridines and Cys231(O). Yellow and black indicate the presence and absence of the H-bond, respectively. (C) Snapshots from the simulation showing representative structures for each of the observed states.

The experimental structure of the complex has only three nucleotides (U1–U3) resolved with the middle U2 bound in the pocket. In our simulations of this structure, we observed either the U1 or U3 terminal nucleotides eventually entering the pocket as well, forming the vertical or horizontal states (Figure 2). In some cases, U2 would later depart the pocket, leaving U1 or U3 as the single nucleotide bound. This corresponds to sliding of the TbRGG2 RRM along the bound RNA by one nucleotide, raising a possibility of an intriguing stepwise nucleotide shift mechanism with the vertical and horizontal states acting as intermediates. We next performed simulations with extended 5′-UUUUU-3′ bound RNA (see Methods), to explore this potential shift mechanism further. In these simulations, we regularly observed multiple such transitions in either direction, supporting the existence of the spontaneous diffusion-scanning mechanism (Figure 3 and Table 2). In some cases, we were able to observe a shift of the entire bound RNA sequence all the way to the U0 or U4 terminal nucleotides (Figure S3). In conclusion, our simulations strongly suggest that TbRGG2 RRM is capable of rapid linear diffusion along the bound poly(U) RNA by utilizing the vertical and horizontal binding states as intermediates. A shift by a single nucleotide can be completed as quickly as 0.5 μs, counting from the formation of the intermediate. As no biasing force was applied to facilitate the transitions, they randomly proceeded in upstream or downstream directions. The diffusion was further sped up by temporary disruptions of the binding, occasionally leading to no nucleotide being present in the binding pocket (the unbound state). This sometimes allowed the protein to “jump over and skip” some nucleotides by reattaching further up- or downstream.

**Table 2:** Number of Transitions Observed in MD Simulations of the TbRGG2 RRM–Poly(U) Complex

simulation	starting state^a^	5′^b^	3′^b^	simulation length [μs]^c^
Simulations of Bound Complexes				
MD2|R1	[U2]	4	1	5
MD2|R2	[U2]	2	2	5
MD2|R3	[U2]	3	1	10
MD2|R4	[U2]	0	1	10
MD3|R1	[U2=U3]	7	7	10
MD3|R2	[U2=U3]	3	3	10
MD3|R3	[U2=U3]	3	3	2
MD3|R4	[U2=U3]	1	1	2
MD3|R5	[U2=U3]	0	0	2
MD3|R6	[U2=U3]	0	0	2
MD4|R1	[U1=U2]	6	5	10
MD4|R2	[U1=U2]	1	3	10
MD4|R3	[U1=U2]	1	1	2
MD4|R4	[U1=U2]	2	0	2
MD4|R5	[U1=U2]	3	3	2
MD4|R6	[U1=U2]	1	0	2
Spontaneous Binding Simulations				
MD13|R1		3	4	10 (0.35)
MD13|R2		5	4	10 (0.13)
MD13|R3		9	8	10 (0.95)
MD13|R4		4	3	10 (0.18)
MD14|R1		1	1	10 (0.81)
MD14|R2		7	9	10 (0.44)
MD14|R3		8	8	10 (0.41)
MD14|R4		0	0	10 (−)
MD15|R1		0	0	10 (−)
MD15|R2		0	0	10 (0.05)
MD15|R3		5	5	10 (0.02)
MD15|R4		0	0	10 (1.20)
MD15|R5		4	3	10 (5.59)
MD15|R6		0	0	10 (−)

a Type of binding pocket interaction in the initial structure of the simulation. Simulations MD13–MD15 were spontaneous binding simulations, with the two molecules completely separated at the start.

b Number of transitions in upstream or downstream (5′ and 3′) directions; it refers to any change of the identity or number of uridines occupying the binding pocket. For the spontaneous binding simulations, this was counted once the first successful binding attempt was observed.

c For spontaneous binding simulations, the number in parentheses refers to the time of the first successful binding. When no number is stated, no successful binding was observed on the simulation time scale.

### Cytidine Tends to Be Rejected by TbRGG2 RRM during the Diffusion Process

**Figure 4:**
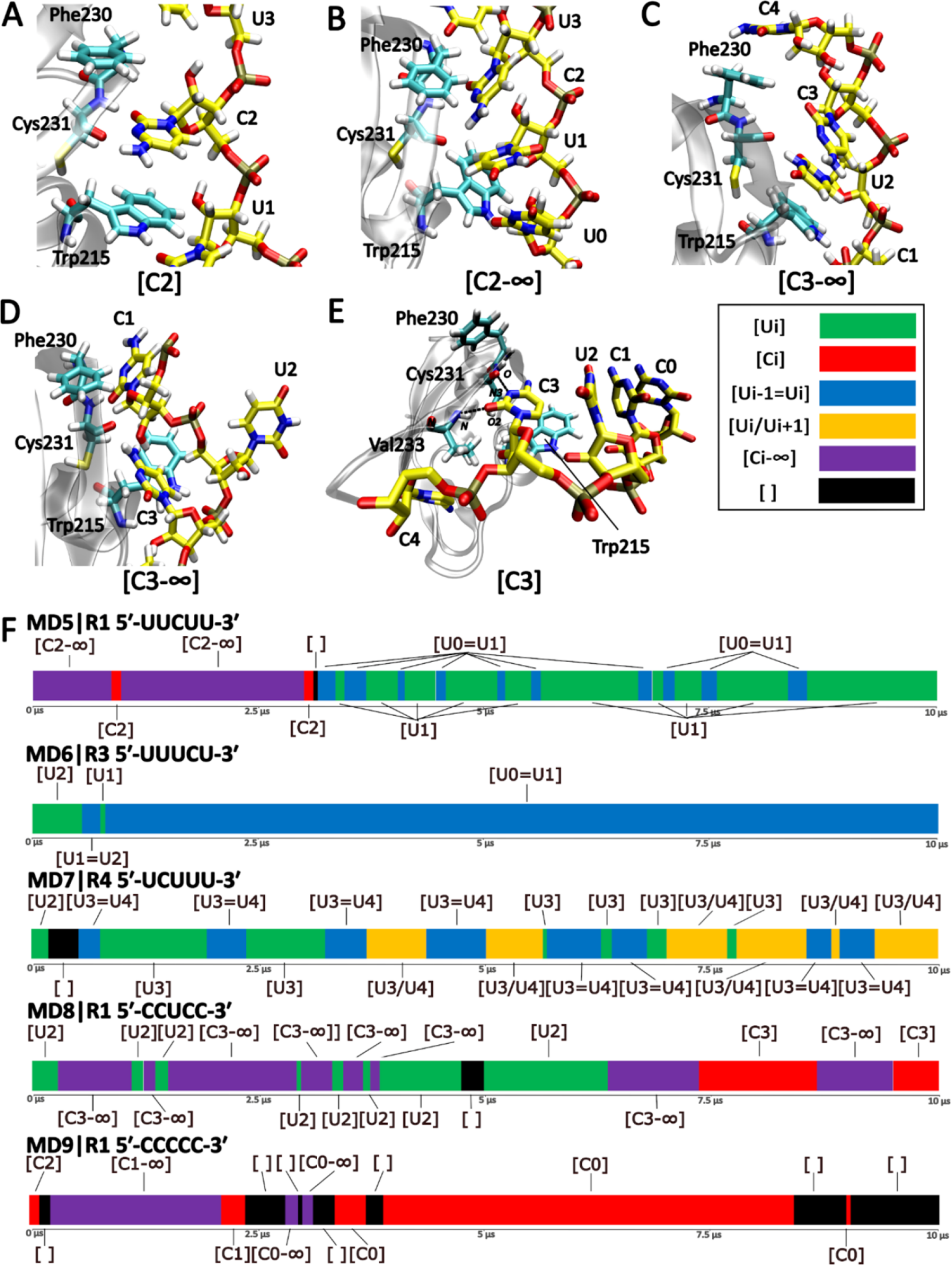
Simulations of the TbRGG2 RRM bound to cytidine-containing RNAs. (A) Starting structure of MD5 simulations with 5′-UUCUU-3′ bound. (B, C) Two variants of the hybrid state, where uridine and cytidine simultaneously occupy the pocket. (D) State with only the backbone of the cytidine entering the binding pocket. (E) One of the arrangements attempted by the system to form an interaction with the cytidine. Due to the enormous structural richness of the binding states involving cytidine, we collectively label all of them (including the hybrid states) as “Ci–∞,” referring to a variety of states in which the protein tries to achieve stable binding of the cytidine by simultaneously forming interactions with uridine, another cytidine, or part of the RNA-backbone. Binding of a single cytidine is labeled as Ci. (F) Time development of the binding pocket occupancy in the individual cytidine-containing systems (MD5–MD9). A single representative simulation of each system is shown (see Figure S3 for the rest).

Above, we have described the mechanism that allows TbRGG2 RRM to quickly diffuse along the poly-(U) RNA (Figure 3). To determine how the RRM would respond to an encounter with a non-uridine nucleotide, we explored multiple systems where selected uridines of the bound 5′-UUUUU-3′ RNA were mutated to cytidines (see Methods). First, we explored the 5′-UUCUU-3′ system, making the C2 nucleotide bound in the pocket at the simulation start. This mutation abolishes the critical Cys231(O)–Ui(N3) H-bond, causing an immediate misalignment of the cytidine with respect to the Trp215 and Phe230 side chains (Figure 4). Soon after the start of the simulation, one of the neighboring uridines entered the pocket, forming a state best described as a hybrid state, a combination of the vertical and horizontal states (Figure 4 and Supporting Information). In all 5′-UUCUU-3′ simulations, the initially bound cytidine was ultimately irreversibly expelled from the binding pocket and supplanted by neighboring uridines (Figure S3).

Second, we explored the effect of placing cytidine immediately upstream or downstream of the single bound uridine. We subsequently observed attempted shifts in both directions, but those toward the cytidine almost universally failed, resulting ultimately in the RNA shifting toward the uridine-rich portion of the RNA. In other words, in the 5′-UCUUU-3′ systems, the RNA tended to shift toward the 3′-end while the opposite occurred for the 5′-UUUCU-3′ simulations. In one case, we observed the RNA separating completely from the protein during an attempted shift in the direction of the cytidine, followed by rebinding further along the RNA chain, thus entirely skipping the non-native cytidine. Third, we tried mutating all of the uridines except U2 to cytidines, i.e., the 5′-CCUCC-3′ sequence, thus potentially making the shifts unfavorable in both directions. Indeed, we observed the U2 located in the binding pocket being regularly invaded by the flanking C1 or C3 (Figure 4C,D). However, attempts to occupy the binding site by a cytidine were unstable, followed by either quick restoration of the sole U2 binding or dissociation of the RNA from the binding pocket. For the sake of completeness, we also explored the behavior of the 5′-CCCCC-3′ system. In this case, the system initially tried to explore numerous conformational arrangements involving the cytidines; however, none of them were stable, and the RNA eventually left the binding pocket entirely.

### Specific Protein Mutations Hamper the Diffusion Mechanism

**Figure 5:**
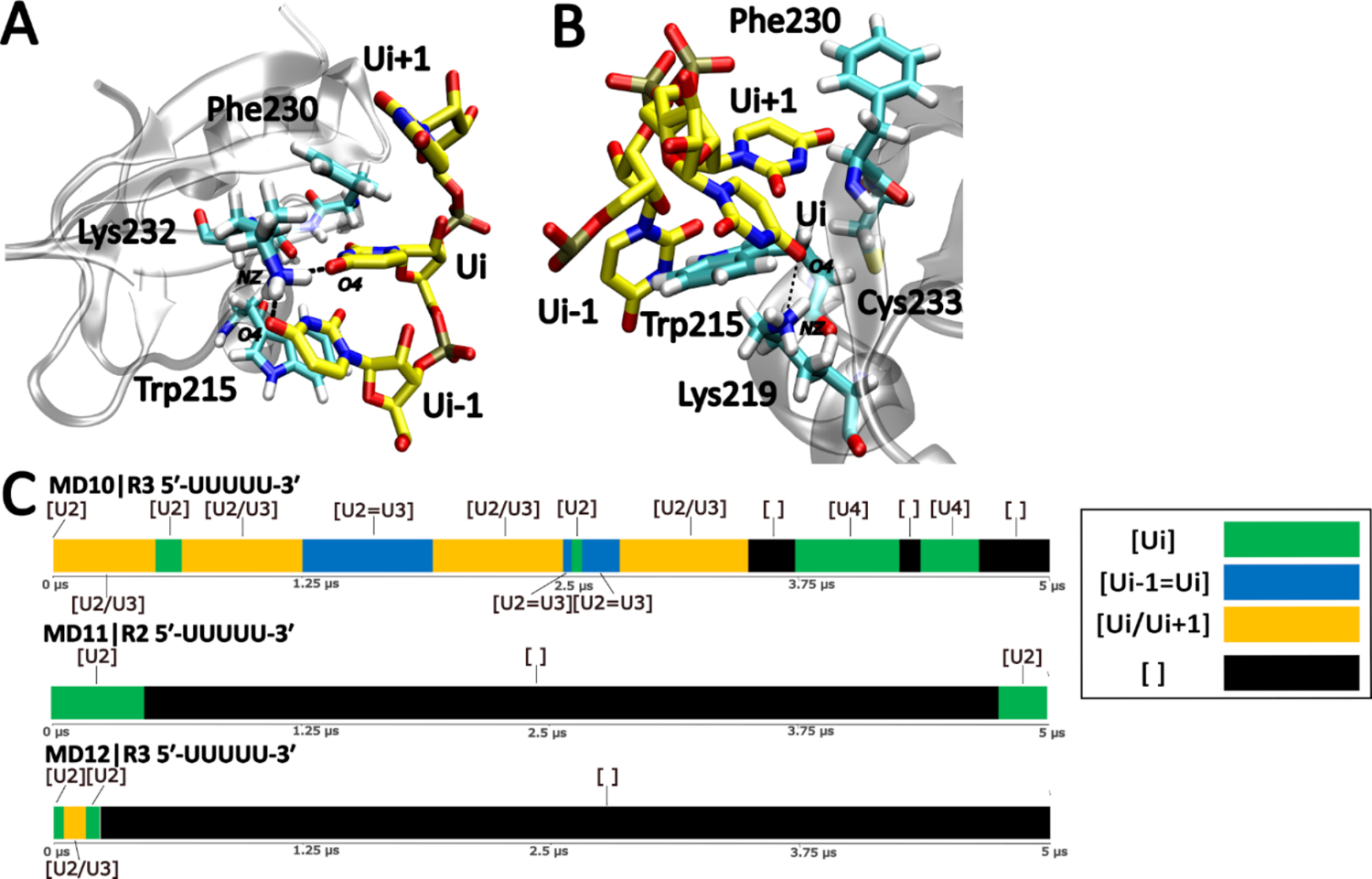
Role of the Lys232 and Lys219 residues vicinal to the binding pocket. (A) H-bonds formed between Lys232 and the uridines in the vertical state. (B) H-bond between the Ui+1 nucleotide in the horizontal state and Lys219. For clarity, not all of the nucleotides are displayed. (C) Time-development of the binding pocket occupancy in selected simulations with mutated proteins. A single representative simulation of each system is shown (see Figure S3 for the rest).

In our simulations, we observed the side chain of Lys232 influencing the dynamics and orientation of the uridines occupying the binding pocket by H-bonding with the Ui(O4) atoms (Figure 5A). Similarly, the Lys219 side chain located on the opposite side of the binding pocket from Lys232 often formed H-bonds with the Ui+1 nucleotide of the horizontal state (Figure 5B). To determine how the Lys232 and Lys219 residues impact the characteristic dynamics of the TbRGG2 RRM binding pocket and the diffusion-scanning mechanism, we prepared complexes in which they were replaced by alanines. The Lys232Ala (see Table 1 for the list of simulations) mutation led to increased flexibility of the nucleotide in the binding pocket, and the diffusion mechanism was essentially abolished, with only a single greatly unstable vertical state observed (Figure S3). The uridines in the binding pocket could still be replaced by adjacent uridines, but these replacements occurred solely via temporary dissociation of the RNA from the binding pocket, followed by a new binding. In conclusion, the simulations suggest that the Lys232 residue is important for properly aligning the nucleotides in the binding site as well as enabling the diffusion mechanism.

On the other hand, simulations with the Lys219Ala mutant revealed a behavior similar to the wild-type, except for slightly increased dynamics of the horizontal states and altered populations of some of the H-bonds. With both mutations combined, the same behavior as with the Lys232Ala substitution was observed.

### Spontaneous Binding Simulations Detail the Involvement of Residues Proximal to the Binding Pocket

**Figure 6:**
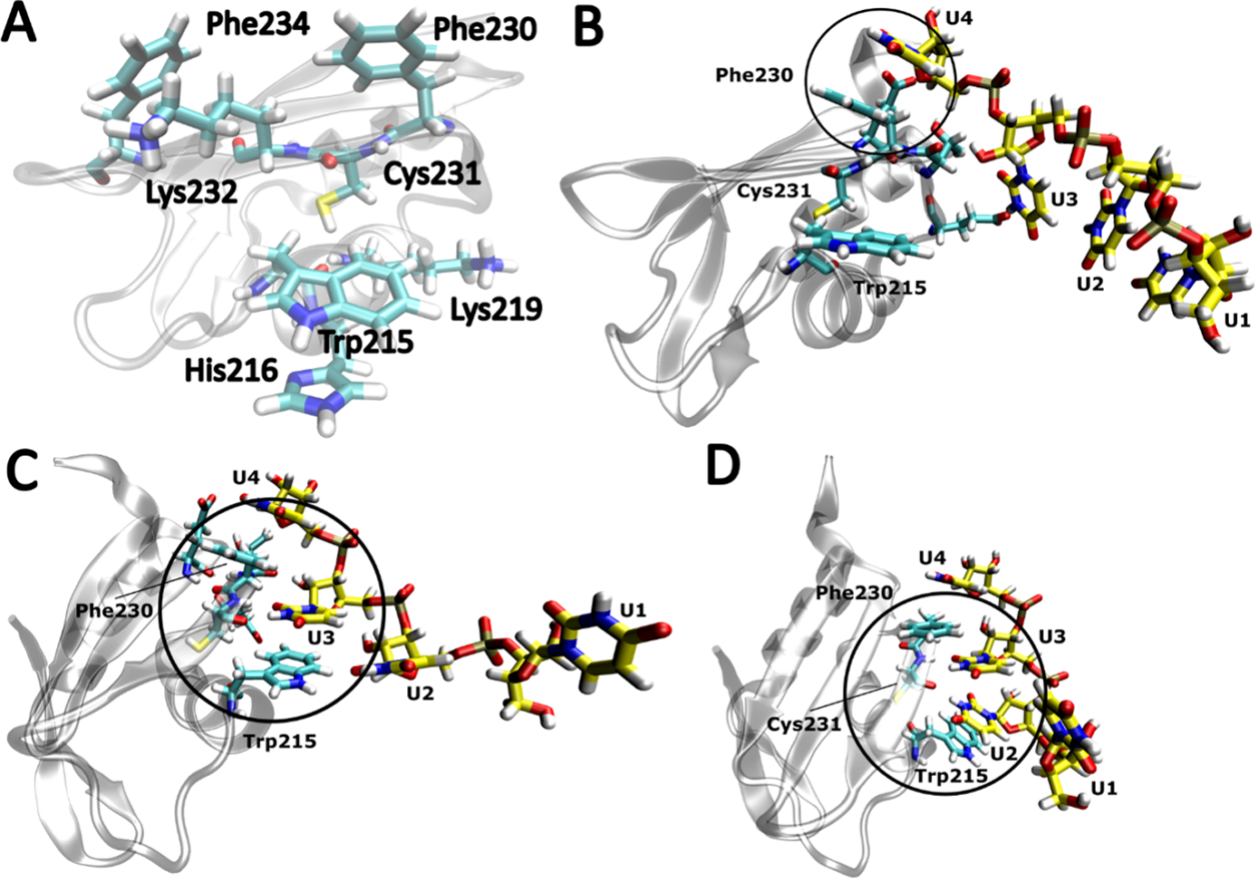
Example of the binding pathway of poly-(U) RNA to TbRGG2 RRM. Upon coming into proximity of the protein residues in the vicinity of the binding pocket (A), stacking interactions formed between the RNA and these residues (B), shackling the RNA in this location and giving it time to gradually establish the native protein–RNA interface (C). (D) Vertical state formed later on.

We have carried out a set of simulations starting from an entirely unbound state (see Methods and ref (23)). As observed earlier for other RRM-ssRNA complexes,^23^ the RNA typically made first contact with an arbitrary surface residue before later spontaneously moving toward the native binding site. As the RNA approached the binding area, the uridines formed stacking interactions with residues proximal to the binding pocket (Figure 6A,B) and H-bonds and salt bridges with Lys232, which appeared to guide and preorganize the RNA for binding. At the end of the binding process, a single uridine would occupy the binding pocket (Figure 6C). Subsequently, we observed the same equilibrium dynamics involving single uridine, horizontal, vertical, and unbound states, as seen in the simulations started from the fully bound complexes (Figure 6D). The protein also diffused along the RNA using the same mechanism as described above. We have observed more RNA dissociation events in our spontaneous binding simulations than in the simulations starting from the fully bound complex, a consequence of having used the poly-(U) tetramer instead of the pentamer (see Methods). This dissociation usually involved the RNA leaving the binding pocket but still interacting with the proximal amino acid residues, essentially reversing the steps of the association process (see above). In other words, the uridines left the binding pocket, but the RNA maintained its molecular contact with the protein surface. More rarely, the RNA would completely depart from the protein surface. In both cases, new binding attempts typically followed.

## Discussion

### Equilibrium Dynamics Is Integral to TbRGG2 RRM Recognition of Poly-(U) RNA

We have used unbiased MD simulations with the OL3-Stafix RNA and ff12SB protein ffs to explore the binding of the TbRGG2 RRM domain to its poly-(U) RNA substrate. We use the term “unbiased MD” because, although Stafix biases the ensemble against spuriously compacted RNAs, it does not restrict the dimensionality of the protein–RNA interaction or the binding process in the way predefined collective variables would. In addition, while the Stafix modification was developed to address specific types of RNA MD simulation tasks, it is not limited to particular systems. We have carried out simulations starting from the experimentally determined (X-ray crystallography) structure as well as simulations starting from an entirely unbound state where the RNA and protein are separated. The results show good convergence, with qualitatively the same dynamics observed from both starting states. According to the experimental structure,^41^ the interface of the complex consists of a single binding pocket recognizing a single uridine. Such a minimalistic interface is rather unusual among the RRM domains, which typically contain multiple binding pockets, recognizing several consecutive nucleotides.^3^ It was suggested that homodimerization of two TbRGG2 RRM domains compensates for its minimalistic interface.^41^ However, even two binding pockets selective for uridines acting in tandem seem at first sight unlikely to achieve the necessary specificity and affinity, which is in a micromolar range. Based on the MD simulations, we suggest that TbRGG2 RRM utilizes extensive equilibrium dynamics to compensate for its solitary binding pocket. First, the simulations clearly confirm that the TbRGG2 RRM contains just one binding site specifically recognizing uridines. The binding site is well reproduced in the fully bound simulations, showing the uridine forming base-specific interactions with the protein backbone, with the nucleobase sandwiched between Trp215 and Phe230 aromatic side chains (Figure 2). The native RNA-binding site is readily located also in our spontaneous binding simulations (Figure 6). Several amino acids proximal to the binding site were found to be engaged in the binding process, helping to navigate the RNA to the native position and stabilizing the temporarily (partially) unbound states (Figure 6). Note that for fast initial binding, it could be advantageous to have just one binding pocket, as proteins with several pockets are more sensitive to the precise positioning of the incoming RNA and can have multiple binding registers that need to be explored.^23^ We have carefully inspected the RRM-RNA complexes available in the PDB database (∼130 structures; see the Supporting Information). However, we did not identify any other RRM protein–RNA complexes with a similarly structured interface. Such complexes might be underrepresented due to their higher interface dynamics and the challenges this poses for determination using structural biology methods. In general, the protein–RNA complexes represent a carefully orchestrated evolutionary compromise between affinity, specificity, and substrate exchange rates. The individual proteins need to be able to unambiguously recognize their specific target sequences in the vast pool of cellular RNAs (specificity), and they need to be able to hold onto them for a sufficient amount of time (affinity) to accomplish their biological function. An often underappreciated factor is that the proteins also need to be able to achieve all of this on biologically relevant time scales, necessitating a swift discarding of noncognate and near-cognate RNAs encountered in the pool. The inevitable presence of numerous near-cognate RNA sequences limits how strong the protein–RNA affinity can get before slow substrate exchange starts hampering its biological purpose. Even once the protein encounters the target RNA, it needs to locate its specific cognate binding motif(s)/sequence(s) among potentially hundreds of nucleotides.

### Unique Equilibrium Dynamics of the TbRGG2 RRM Complex Effectively Circumvents Limitations of Stochastic Diffusion

The simulations of TbRGG2 RRM reveal a unique structural mechanism through which all of the contradictory requirements placed on protein–RNA complexes can be elegantly reconciled and balanced. Namely, in addition to the single uridine binding state (shown by experiments), we have observed two additional binding states with two uridines simultaneously occupying the binding pocket, referring to these states as vertical and horizontal states (Figure 2). Formation of these additional states is facilitated by only a modest widening of the binding pocket by up to ∼1.0 Å. All three states are engaged in fast μs-scale interconversion dynamics. The RNA can also temporarily unbind from the binding pocket, transiently interacting with the residues proximal to the binding pocket before reentering the pocket. More rarely, the RNA left the protein surface, which was followed by a new binding attempt. The interconversion dynamics among the three principal binding (sub)states allows a smooth exchange of the uridines in the binding pocket, facilitating a low-barrier and virtually unobstructed diffusion of the RRM along the whole poly-uridine sequence. Such a recognition mechanism is uniquely suited for a solitary binding pocket as more pockets would require the bound RNA to shift its position in all pockets in a concerted manner. When lacking external energy provided, e.g., by ATP hydrolysis, such RNA shifts are accomplished strictly by stochastic movements.^59^ The traditional models of purely stochastic diffusion envision the protein step-by-step shifting along the nucleic acid in a linear fashion or repeatedly binding and unbinding at different sites set apart by a few to hundreds of nucleotides until reaching the cognate binding motif. However, the strict application of both of these models leads to biologically unsustainable rates of exchange and recognition.^60^ The presence of a single binding pocket considerably lowers the energy barriers and elegantly circumvents the inherent limitations of stochastic diffusion.

**Figure 7:**
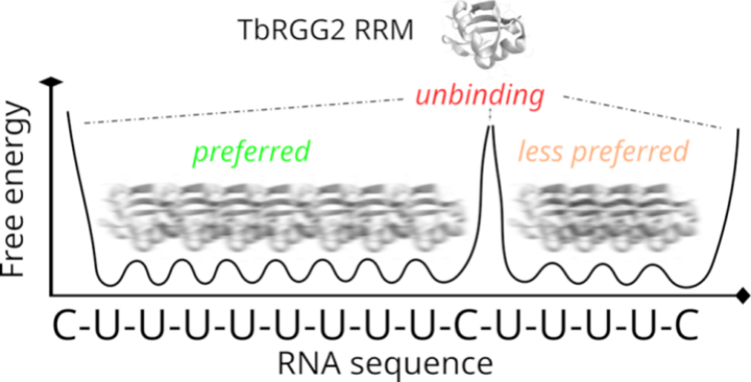
Scheme of the suggested equilibrium dynamics of the TbRGG2 RRM bound to U-rich RNA. The flexible binding pocket allows low-barrier transitions along the continuous poly-(U) regions of the RNA on the microsecond time scale at physiological temperatures, where it will essentially form a single flat, mildly undulated basin on the energy landscape. The fuzzy protein images above the individual poly-(U) stretches indicate the protein is rapidly shifting within the region. Meanwhile, the encounter with non-uridine nucleotide destabilizes the complex and can lead to unbinding and another binding attempt. We suggest this mechanism allows the protein to quickly locate the longest stretches of uridines, which then become the preferred binding sites by entropic effects. Utilization of only a single binding pocket ensures noncognate substrates are rapidly discarded, while the entropically advantageous equilibrium dynamics accessible only for the cognate sequence guarantees high specificity. The one-dimensional free energy plot is merely illustrative, being deduced from the simulation analyses; it is not a result of any direct measurements or computations.

In summary, our unbiased MD simulations indicate that the free-energy landscape of the bound TbRGG2–poly(U) complex is very broad (flat) and consists of a set of shallow states (or substates) separated by small free-energy barriers, so that they interconvert on the μs time scale. Such a structure of the free-energy surface allows fast movements of TbRGG2 RRM along the whole poly(U) ssRNA sequence and may contribute entropically to the binding affinity. We suggest the entire poly-(U) stretch where the RRM is bound essentially corresponds to a single continuous binding region at 300 K (Figure 7). Upon encountering non-native nucleotides like cytosines, TbRGG2 RRM is unable to stably accommodate them via any of the three binding states observed (Figure 2), causing the protein to revert back to the uridine-rich regions. Alternatively, it can “skip” the cytidine by the RNA temporarily leaving the binding pocket and rebinding further up or downstream. In this fashion, the TbRGG2 RRM is supremely tuned for rapidly locating the most U-rich region of any RNA sequence it encounters. Although the qualitative details of the transition mechanism are evident from our MD simulations, we did not attempt to create any quantitative free-energy model by comparing the different substates. Among other reasons (such as the force-field approximation), the populations of the substates varied rather significantly among the replicate simulations, highlighting the fact that the populations are not quantitatively converged (Table S1 and Figure S4).

### Spontaneous Binding Simulations Demonstrate the Role of the Residues Proximal to the Binding Pocket and Confirm the Unique Equilibrium Dynamics

After a random first contact with any part of the protein surface, the binding process commenced with the RNA approaching the hydrophobic or positively charged residues proximal to the binding pocket (Figure 6A), forming nonspecific interactions with them. In simulations started from a fully bound complex, the same residues would bind the RNA when it temporarily left the binding pocket. The protein segments surrounding the binding site might be guiding or funneling the complex toward its free-energy minimum. Such nonspecific contacts are similar in principle to the “pre-binding state” observed in MD simulations of the HuR RRM3,^23^ although less structurally defined. Upon establishing the native binding, our spontaneous binding simulations showed qualitatively identical equilibrium dynamics as the simulations started from a fully bound complex, confirming the results are not overly dependent on the starting structure. At the same time, the spontaneous binding simulations suggested that the vertical and horizontal binding modes are not part of the binding–unbinding landscape of the complex, as no binding events progressing via these substates were observed. Instead, they appear to be distinct conformational states associated with the low-barrier equilibrium dynamics of the fully bound complex. We note that in an effort to achieve a greater success rate of the spontaneous binding, we tested several ff setups, including a lower Stafix factor (0.5 vs 0.1),^23^ a different water model (SPC/E vs OPC),^48,49^ and the use of gHBfix19^50^ potential function (see Methods). However, these setups resulted in no qualitative variation in the resulting ensembles. Importantly, identical binding mechanisms were observed across all of the tested protocols. The MD15 ensemble, overall, showed fewer successful binding attempts (Supporting Information), which we tentatively attribute to the use of a Stafix factor of 0.1, potentially excessively disrupting the vertical and horizontal states. It is worth noting that a Stafix factor of 0.5 has been shown to be sufficient to achieve the full benefits of the Stafix modification while minimizing the side effects.^23^ Lastly, we have also attempted spontaneous binding simulations of the poly-(G) RNA. The poly-(G) sequence is known to be bound by TbRGG2 RRM through a binding site distinct from the poly-(U).^41^ However, the bound structure showing poly-(G) RNA binding is not available. Unfortunately, we were unable to make any conclusions from our spontaneous binding simulations (not shown). We speculate the binding of poly-(G) sequence may involve some hitherto unknown conformational change of the protein that was not sampled on our simulation time scale.

### Dimerization of TbRGG2 RRM Combined with Its Equilibrium Dynamics Could Further Enhance the Selectivity for U-Rich RNA Sequences

TbRGG2 possesses a dimerization domain located C-terminal of the RRM.^41^ However, the dimerization domain has not been included in the experimentally determined X-ray structure of the RRM (PDB: 6E4P), and while the RRMs are making molecular contacts in the asymmetric unit, it is not directly stated that these correspond to the native dimers.^41^ In fact, it is unlikely, as the relative positions of the RRMs within the crystal lattice do not allow straightforward addition of the dimerization domains by molecular modeling. Furthermore, our simulations of the putative RRM homodimers found in the crystal lattice, both with and without the RNA bound, led to almost instantaneous and permanent loss of the experimental protein–protein interface (Figure S5). The RRMs remained in some molecular contact but failed to establish any interface that would be stable (Supporting Information). Even in the absence of the C-terminal dimerization domains, such swift loss of native interface interactions should not occur in MD simulations for even very weakly bound dimers, especially considering that the ff generally overstabilizes the solute–solute interactions.^23^ We therefore conclude the arrangement and interactions of the TbRGG2 RRMs, as observed in the experimental structure, reflect the constraints of the crystal lattice rather than a preference for a particular dimer interface. Multiple protein chains tightly surrounding and binding the single RNA molecule might also be what had stalled the RNA diffusion mechanism and allowed for structure resolution. This suggests that the TbRGG2 homodimerization is driven solely by the C-terminal domains, placing the RRMs in close proximity but with no major interface interactions between them. The equilibrium dynamics of the RRM-ssRNA interface observed in this work can then be expected to independently occur for both binding pockets of the two RRMs also within the homodimer context of full length TbRGG2. With two RRMs acting like independent “walkers” along the RNA sequence, the specificity of TbRGG2 for U-rich parts of the RNA sequences would be vastly increased.

## Conclusions

Using large-scale standard atomistic molecular dynamics simulations, we showed that the TbRGG2 RRM binding site for poly-(U) RNAs is characterized by low-barrier interconversion dynamics involving three distinct binding substates, allowing a smooth exchange of the uridines in the binding pocket. All three binding substates are highly selective for uridines, and we observed completely spontaneous diffusion of the poly-(U) sequences all the way to the strand termini. The presence of non-native cytidines tends to cause either a swift transition back toward the U-rich regions or eventual disengagement of the complex, followed by another binding attempt elsewhere on the sequence. Using this mechanism, the TbRGG2 RRM should be able to rapidly locate the most uridine-rich part of any RNA sequence. Spontaneous binding simulations, initiated with the two molecules fully separated, reveal qualitatively identical behavior, confirming that the observed equilibrium dynamics of the fully bound complex is not part of the binding-unbinding dynamics. Dynamical binding mechanisms and sites such as the one employed by TbRGG2 RRM could constitute a so far underappreciated element of protein–RNA recognition, difficult to detect by conventional structural biology experiments but with potentially high utility in the astonishingly diverse world of protein–RNA complexes.

## Data Availability

All of the data necessary to support and reproduce the findings of this study are available in this text and in the Supporting Information. The data necessary to independently reproduce the MD simulations and the trajectories discussed have been deposited with Zenodo (accession code: 13929049). The AMBER package can be licensed and downloaded from AMBER (http://ambermd.org/). The PyMOL can be licensed from Schrodinger (https://pymol.org/). The VMD molecular visualization program can be licensed from UIUC (http://www.ks.uiuc.edu/Research/vmd/). Python scripts to implement Stafix for topology files in AMBER and Gromacs can be downloaded from Zenodo (https://zenodo.org/records/7273724).

## Supporting Information Available

Additional information on Stafix; simulations of the putative TbRGG2 RRM homodimers; additional information on the intermolecular H-bonds formed by single; vertical and horizontal binding states; comments on the binding states formed by cytidines; and supporting figures, graphs, and tables (PDF)
